# Molecular mechanisms of MYCN-dependent apoptosis and the MDM2–p53 pathway: an Achille’s heel to be exploited for the therapy of MYCN-amplified neuroblastoma

**DOI:** 10.3389/fonc.2012.00141

**Published:** 2012-10-12

**Authors:** Marialaura Petroni, Veronica Veschi, Alberto Gulino, Giuseppe Giannini

**Affiliations:** Department of Molecular Medicine, University “La Sapienza”Rome, Italy

**Keywords:** Neuroblastoma, HIPK2, MYCN, HMGA1, MDM2-antagonists

## Abstract

The p53 oncosuppressor is very seldom mutated in neuroblastoma, but several mechanisms cooperate to its functional inactivation in this tumor. Increased MDM2 levels, due to genetic amplification or constitutive inhibition of p14^ ARF^, significantly contribute to this event highlighting p53 reactivation as an attractive perspective for neuroblastoma treatment. In addition to its role in tumorigenesis, MYCN sensitizes untransformed and cancer cells to apoptosis. This is associated to a fine modulation of the MDM2–p53 pathway. Indeed MYCN induces p53 and MDM2 transcription, and, by evoking a DNA damage response (DDR), it stabilizes p53 and its proapoptotic kinase Homeodomain Interacting Protein Kinase 2 (HIPK2). Through the regulation of the HIPK2-p53 inhibitor High Mobility Group protein A1 (HMGA1) and the homeobox proteins BMI-1 and TWIST-1, MYCN establishes a delicate balance between pro- and antiapoptotic molecules that might be easily perturbed by a variety of insults, leading to cell death. MDM2–p53 antagonists, such as Nutlin-3, are strikingly prone to inducing death in MYCN-amplified neuroblastoma, by further pushing on HIPK2 accumulation. Here we discuss implications and caveats of exploiting this pathway and its connections to MYCN-induced DDR for a tailored therapy of MYCN-amplified neuroblastoma.

Neuroblastoma, one of the most common tumor of childhood, is an extraordinary challenging nosocomial entity due to its tremendous heterogeneous behavior ranging from spontaneous regression to very aggressive and inexorable progression. In 2009, the International Neuroblastoma Risk Group defined seven parameters (stage, age, histologic category, grade of tumor differentiation, status of MYCN oncogene, chromosome 11q status, and DNA ploidy) required to stratify neuroblastoma patients into tentatively homogeneous risk groups and estimated that the high-risk fraction (5-year event free survival <50%) accounted for about 40% of their 8800 patients (Cohn et al., 2009). About half of the high-risk neuroblastoma patients are characterized by the MYCN amplification (MNA) status, which represents the most relevant independent negative prognostic factor in neuroblastoma. Indeed MNA occurs in about 20–25% of the cases and is most frequently associated to treatment failure. Supporting its pivotal biological role in neuroblastoma, MYCN overexpression in the neural crest is sufficient to promote the development of neuroblastic tumors in transgenic mice (Weiss et al., 1997). Consistently, inhibition of MYCN leads to death, differentiation, and/or impairment of cell growth, suggesting that knocking it out in MYCN-addicted tumors might represent a very effective strategy to cure this subset of tumors. Unfortunately, however, efficacious and immediately translatable methods to deplete MYCN directly into a neuroblastoma patient are not yet available. While many groups are looking for strategies aimed at directly targeting Myc (Soucek et al., 2008; Hogarty and Maris, 2012), recent discoveries opened the possibility to deliver target therapies for MNA tumors based on synthetic lethal approaches (Molenaar et al., 2009; Cole et al., 2011) or by enhancing the anti-cancer pathways intrinsically activated by MYCN. Here we focus our attention on the p53-dependent apoptotic pathway activated by MYCN as a potential target for the treatment of MNA neuroblastoma.

## THE ARF/MDM2/p53 PATHWAY IN NEUROBLASTOMA

p53 is a master oncosuppressor protein involved in protecting cells from genetic instability by inducing cell cycle arrest or apoptosis in response to cellular stress and DNA damage. Its genetic inactivation characterizes more than 50% of human cancers and its functional impairment occurs in most of the remaining part, highlighting the “reactivation of p53” as a treatment option potentially effective for an extremely broad range of human cancers. Indeed, p53 restoration already proved to be effective in promoting tumor regression in animal models and several strategies aimed at restoring p53 function (i.e., p53 gene therapy, wild type p53 restoration via small molecole/MDM2-antagonists and mutant-p53 folding restoration) are being currently tested in clinical trials (Cheok et al., 2011).

p53 is very seldom mutated in childhood cancer of the central and peripheral nervous system and in primary neuroblastoma its mutation rate does not exceed 1–2% (Vogan et al., 1993). However, multiple hits seem to cooperate to p53 functional impairment in this tumor. Its delocalization to the cytoplasm was initially thought to contribute to p53 inactivation in neuroblastoma (Moll et al., 1996). However, the significance of this finding is still controversial, since others have shown p53 properly nuclear in neuroblastoma cells. Moreover, p53 may also instruct cells to undergo apoptosis by directly localizing at mitochondria and its differential localization might influence the balance between apoptosis and autophagy (Maiuri et al., 2010), whose outcome might be rather important for the survival of a cancer cells. Thus, extranuclear p53 should not be considered necessarily inactive and the biological significance of cytoplasmic p53 in neuroblastoma definitely needs to be more thoroughly investigated.

**FIGURE 1:**
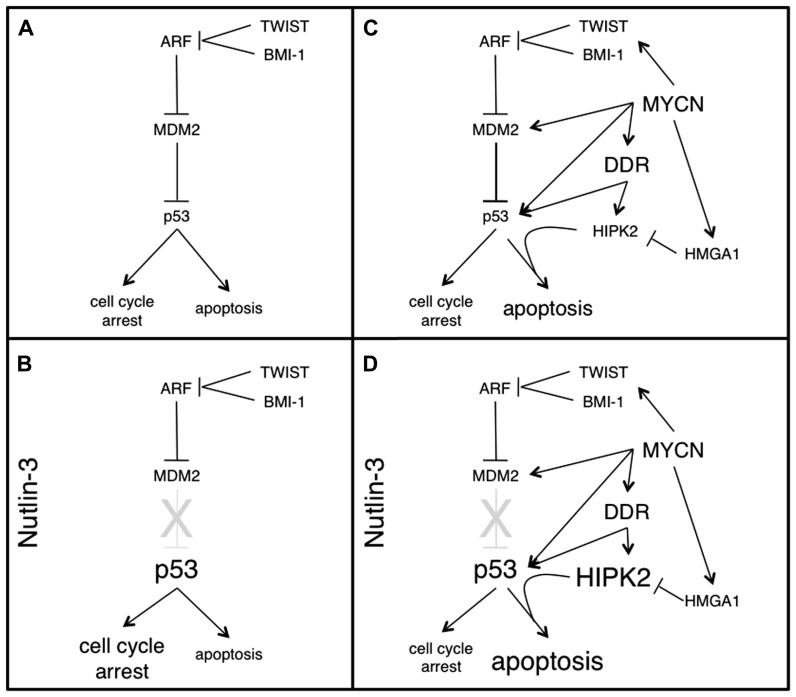
** The MDM2–p53 pathway, the apoptosis-sensitive phenotype induced by MYCN and their modulation by Nutlin-3, in neuroblastoma cells.** p53 is rarely mutated in neuroblastoma. Rather, its inactivation occurs at the functional level, mostly via increased MDM2 activity **(A)**. When p53 is uncoupled from MDM2 (by means of Nutlin-3) an unmodified p53 accumulates in MYCN single copy cells, driving them mostly into a reversible cell cycle arrest **(B)**. MYCN overexpression and MNA amplification lead to dramatic changes in multiple components of the MDM2–p53 pathways, modulating both pro- and antiapoptotic factors in a coordinated fashion, which does not result in cell growth inhibition or apoptosis, but rather in an apoptosis-sensitive phenotype, which strongly depends on p53 and HIPK2 and can be easily recruited by several sources of stress **(C)**. In MNA cells, Nutlin-3 not only uncouples p53 from the MDM2 inhibition, but also leads to strong HIPK2 induction and consequently to commitment of p53 toward apoptosis **(D)**.

Although p53 expression levels may be directly repressed by miRNAs (Swarbrick et al., 2010), deregulation of the ARF/MDM2 pathway appears to be the most biologically relevant and the most “druggable” target contributing to p53 functional inactivation in neuroblastoma cells. The E3 ubiquitin ligase MDM2 is a master regulator of p53 activity. Indeed it mediates p53 ubiquitination and degradation, as well as cytoplasmic delocalization and transcriptional inactivation. Disruption of MDM2–p53 interaction physiologically occurs upon phosphorylation of p53 on serine 15 and serine 20 operated by the ATM and CHK2 kinases, respectively, in response to several types of stress. MDM2 activity is further controlled at multiple levels, including autoubiquitination and degradation as well as by direct molecular interactions. In example, by a direct binding to MDM2, p14^ ARF^ impairs its activity on p53. Interestingly, ARF inactivation (due to deletions or promoter methylation) or MDM2 amplification are frequently found in neuroblastoma cell lines. Analysis of a small series (41 cases) of neuroblastoma tumors documented ARF inactivation or MDM2 amplification at diagnosis in about 30% of the cases, while five of six mutations detected on p53 were not present in the primary lesion, but appeared at relapse or after chemotherapy (Carr-Wilkinson et al., 2010). Although with the limitation of the little statistical power, these data provide support to a number of observations: (i) p53 is only rarely mutated in neuroblastoma (up to 15% after relapse and/or progression) suggesting that neuroblastoma has “an innate requirement for baseline p53 activity (perhaps to resist oncogenic stress)” as stated by Kim and Shohet (2009). (ii) The ARF–MDM2 axis is frequently hit in neuroblastoma cells via direct genetic and epigenetic targeting, eventually resulting in high MDM2 activity. The increased expression of the homeobox proteins BMI-1 and TWIST-1 frequently occurring in neuroblastoma may further contribute to this end (Valsesia-Wittmann et al., 2004; Ochiai et al., 2010). (iii) Uncoupling p53 from its negative regulator MDM2 with small molecules competing for the respective docking sites (such as Nutlin-3, MI-63, and RITA among others) should lead to p53 reactivation, a potentially valuable approach to neuroblastoma therapy. Indeed, Nutlin-3, a cis-imidazoline small molecule that mimics a p53-binding peptide, interferes with MDM2 binding to p53 and proved to be very effective to reduce growth and eventually promote apoptosis in neuroblastoma cells (Barbieri et al., 2006; Van Maerken et al., 2006). Of relevance, p53 reactivation via Nutlin-3 leads to cell cycle arrest in non-cancer cells, which might help protecting them from the adverse effects of chemotherapy, in the case of association therapies. In an elegant preclinical study, Van Maerken et al. (2009) showed that Nutlin-3 not only impairs neuroblastoma growth *in vitro*, and *in vivo*, but also prevents tumor metastasis formation in xenograft models. Interestingly, even vincristine- and doxorubicin-resistant xenografts appeared strongly sensitive to Nutlin-3, suggesting this approach does not suffer from cross-resistance limitations, as long as the target cells have wild type p53. A potential limitation to this strategy comes from the observation that p53 reactivation via MDM2-antagonists such as Nutlin-3 not necessarily cause apoptosis in cancer cells, but it may also determine a transient growth inhibition depending on the cell and molecular context (Rinaldo et al., 2009), with obvious consequences on its possibility to eradicate cancer. Concerning neuroblastoma, consistent data indicate that MNA cells are strikingly sensitive to death induced by MDM2-antagonist, while cell growth inhibition might be the preferential outcome in MYCN single copy neuroblastoma (**Figure 1**). The emerging molecular picture providing a rationale for this is depicted in the next paragraph.

## THE APOPTOSIS-SENSITIVE PHENOTYPE INDUCED BY MYCN AND THE MDM2–p53 PATHWAY: BENEFITS AND CAVEATS OF p53 REACTIVATION STRATEGY

MYCN belongs to a conserved family of bHLHZip transcription factors that coordinate cell proliferation, differentiation, and survival as well as the interactions between cells and their microenvironment, including angiogenesis, invasion, and inflammation. Disruption of either c-Myc or N-Myc in mice leads to embryonic lethality due to growth defects in multiple organs and tissues. Additionally, c-Myc is one of the four essential genetic ingredients that efficiently convert fibroblast into pluripotent stem cells, confirming that Myc proteins play an unparalleled role in the regulation of stemness and cell proliferation. Therefore it is not surprising that direct or indirect deregulation of MYC expression occurs in the vast majority of human cancers.

Primary deregulation of Myc expression or experimentally induced Myc overexpression also leads to apoptosis, largely (though not exclusively) via the ARF/p53 pathway, which initially appeared paradoxical for a prototypic oncogene. However, more recently it has been shown that many oncogenes (and more in general oncogenic stimuli unleashing unscheduled cell proliferation) can trigger a DNA damage response (DDR) most likely connected to the replication stress and certainly involving the signaling cascades prompted by the ATM/ATR kinases, which in turn promote p53 accumulation/activation and apoptosis, cell senescence or stable inhibition of cell cycle progression. The DDR, therefore, has been envisaged as an intrinsic anti-cancer barrier that needs to be disrupted (i.e., via inactivation of the ATM or p53) for cell transformation to proceed (Bartkova et al., 2005; Gorgoulis et al., 2005; Di Micco et al., 2006).

Although MYCN induces replication stress, DDR and genetic instability in neuroblastoma cells (Petroni et al., 2011), p53 mutations occur very rarely even in MNA neuroblastoma and p53 haploinsufficiency does not accelerate neuroblastoma development in MYCN transgenic mice, consistent with a modulated functional inactivation of p53 occurring in MNA neuroblastoma (Weiss et al., 1997). Coherently, although MYCN overexpression may induce apoptosis in cells from the nervous system, this was not so frequently reported in neuroblastoma cells. Rather, it has long been recognized an apoptosis-sensitive phenotype that enhances cell death in response to several sources of stress, including chemotoxic drugs, in MYCN overexpressing and MNA cells, *in vitro* (Lutz et al., 1998; Fulda et al., 1999). This drug-sensitive phenotype remains somehow paradoxical in light of the inexorable behavior of MNA neuroblastoma, which relapse and progress very rapidly (eventually after an initial remission) even despite very aggressive therapies, in the majority of the cases.

Work from different labs, including ours, highlighted an entangled molecular network governing the functional interactions between MYCN and p53, which appears to be largely responsible for the well-known apoptosis-sensitive phenotype induced by MYCN. As a matter of fact, MYCN favors p53 accumulation, by directly promoting its transcription (Chen et al., 2010) but also by increasing p53 serine 15 phosphorylation via a canonical DDR (Petroni et al., 2011). Nevertheless, this does not end up with cell growth inhibition (possibly due to impairment of the G1 cell cycle checkpoint) neither it results in relevant cell death. How is p53 suppressed by MYCN? Slack et al. (2005) initially demonstrated that MDM2 is a MYCN transcriptional target and postulated that its increased expression could functionally limit p53 oncosuppressive functions and thus allow tumorigenesis induced by MYCN. Moreover, MYCN promotes the expression of the homeobox gene BMI-1 (Ochiai et al., 2010), further pushing MDM2 activation, via ARF suppression. MDM2, in turn, may facilitate MYCN expression by promoting its mRNA stabilization and translation (He et al., 2011). Therefore multiple evidences seem to converge on MDM2 being the main character in the story. Consistent with these biochemical data, MDM2 deficiency suppresses MYCN-dependent tumorigenesis in transgenic mice (Chen et al., 2009). Of relevance, MYCN also induces the p53 proapoptotic homeodomain interacting protein kinase 2 (HIPK2; Petroni et al., 2011). HIPK2 binds and modulates p53 at multiple levels: it phosphorylates p53 on serine 46, a modification required to commit p53 toward apoptotic target genes, and promotes CtBP degradation thereby derepressing p53-dependent apoptotic targets such as Bax and Noxa (Puca et al., 2010). HIPK2 is an unstable protein whose levels are strictly controlled by the ubiquitin/proteasome system via multiple E3 ubiquitin ligases highly responsive to cellular stress. Consequently, its degradation is suppressed in stressing conditions and HIPK2 accumulates and may eventually elicit its apoptotic potential. In example, upon DNA damage ATM/ATR kinases phosphorylate the Siah-1 ubiquitin ligase loosening its affinity for HIPK2 and promoting its accumulation (Winter et al., 2008). Coherent with this model, MYCN-induced DDR is responsible for HIPK2 accumulation in MYCN overexpressing cells (Petroni et al., 2011). HIPK2 and p53^ S46^ phosphorylation are required for the MYCN-dependent apoptosis-sensitive phenotype both in MYCN overexpressing and in MNA cells (Petroni et al., 2011), indicating that its proapoptotic function is recruited in these cells upon additional stress. Under basal conditions, however, HIPK2 activity might be partially hampered by cytoplasmic sequestration due to the increased expression of the High Mobility Group protein A1 (HMGA1), an additional transcriptional target of MYCN (Giannini et al., 2005; Pierantoni et al., 2007). Therefore, MYCN upregulates a series of molecules impinging on the MDM2–p53 pathway that create a delicate equilibrium between pro- and antiapoptotic factors. Incoming stress, such as exposure to chemotoxic drugs, induces a reversible unbalance by increasing the stoichiometric weight of p53 and HIPK2, which accumulate, bringing up apoptosis in a large number of cells (**Figure 1**), unless MDM2 increase due to p53 transcriptional activation restores the balance, progressively silencing the pathway. The MYCN-dependent apoptotic-sensitive phenotype observed *in vitro* is mirrored *in vivo* by a high level of mitosis-karyorrhexis and by the very frequent initial response to the induction therapy observed in MNA neuroblastoma patients. Nevertheless, MNA tumors most frequently relapse, suggesting that several MNA cells find the way to escape apoptosis and are able to quickly reconstitute local tumor bulk and distant metastatic dispersion. While the increase in p53 mutation rate in relapsed and post-chemotherapy neuroblastoma indicates that inactivation of p53 is relevant for tumor progression, the small amplitude of the phenomenon (up to 15% mutation rate) confirms that the power of the p53 pathway to induce death under treatment is made functionally inefficient, at least in some cells. Thus, although the transient disequilibrium described above justifies the increased rate of apoptosis in MNA cells, it does not translate in a benefit for the patients, rather it pushes the selection of most resistant cancer clones. It is reasonable to believe that a more potent and more permanent unbalancing of the pathway leading to an increased activation of the proapoptotic components (i.e., p53 and HIPK2) could improve the outcome possibly also *in vivo*. Consistently, uncoupling p53 from MDM2, via small molecules MDM2-antagonists, results in massive cell death in MNA neuroblastoma cells either alone or in combined treatment with chemotoxic drugs (Gamble et al., 2011; Petroni et al., 2011). Moreover, Nutlin-3 oral administration efficiently impaired tumor growth and metastatic spread in xenograft models of MNA neuroblastoma (Van Maerken et al., 2009). This enhanced power is likely to be due to its extra-activity on HIPK2, which further accumulates upon treatment and is strictly required for apoptosis to occur in MNA neuroblastoma (Petroni et al., 2011). This is rather unexpected since accumulated and Nutlin-3-bound MDM2 has been shown to maintain its E3 ubiquitin ligase activity and to target HIPK2 for degradation, preventing apoptotic activation of p53 and committing it toward cell growth inhibition, which is a rather frequent outcome in non-hematological cancer cells (Rinaldo et al., 2009). These findings raised arguments that will need to be carefully addressed and whose relevance is not necessarily restricted to neuroblastoma. (a) Depending on the cell context, MDM2–p53 antagonists (such as Nutlin-3) may have opposite effects on HIPK2 and this might drive the biological outcome (i.e., cell growth inhibition versus apoptosis) of p53 reactivation. (b) The molecular mechanisms leading to HIPK2 induction by Nutlin-3 in MNA cells are currently unknown and a better understanding of this issue might help identifying biomarkers predicting the biological outcome of p53 reactivation, with obvious impact on the use of this drug in cancer treatment. (c) The behavior of other p53 reactivating molecules, such as MI-63, or RITA (that differs from the others since it binds the p53 moiety) with respect to the balance between MDM2, p53, and HIPK2 is currently unknown, but there is reason to believe they might do different than Nutlin-3, perhaps also depending on the cell/tumor context (Grinkevich et al., 2009; Rinaldo et al., 2009). (d) Overall our current understanding of the molecular pathways modulating the biological effects of p53 reactivation is rather poor and the expansion of this area of investigation appears mandatory. In example, p53 might promote apoptosis by direct localization at the mitochondria independent of its transcriptional activity and this function might be also activated by Nutlin-3. Consistently, Galectin-3 (another component of the p53 circuitry) impairs mitochondrial apoptosis in neuroblastoma and its repression by Nutlin-3 is required for full-blown apoptosis in MNA cells (Veschi et al., 2012). Furthermore, Nutlin-3 binds and inactivates also Bcl-2 and some of its family members (Shin et al., 2012), with potentially p53-independent effects on mitochondrial membrane permeability. The role of p53 and its molecular circuitry at the mitochondria has not been carefully ascertained in neuroblastoma cells, but further investigation appears necessary for a better understanding of the effects of MDM2–p53 antagonists in neuroblastoma and might help identifying potential biomarkers (in addition to p53 mutational status) to be used for a MDM2–p53 antagonists tailored therapy.

While all of the above supports the hypothesis that the pharmacological reactivation of p53 might be an interesting therapeutic opportunity for MNA neuroblastoma, that further improving the molecular understanding of this pathway is also required to avoid that an oversimplification of the picture might cause unforeseen effects in patients. The increasing levels of complexity in the regulation of the pathway also call into play MDMX as a relevant modulator of p53 mono- or polyubiquitination by MDM2, which would alternatively cause its transcriptional inhibition and nuclear export versus proteolytic degradation, with relevant consequences on the biological outcome in normal and cancer cells (Wang and Jiang, 2012). Nutlin-3-bound MDM2 was shown to retain several activities, including MDMX E3 ubiquitin ligase activity, but the consequence of this in neuroblastoma are completely unexplored. Furthermore, MDM2 interacts with multiple partners and exploits oncogenic (but also oncosuppressive) properties independent of p53 (Manfredi, 2010). Thus far, very little is known about how much these activities are conserved by Nutlin-3-bound MDM2 or by other MDM2–p53 antagonists. Thus, the possibility that unwanted activation of MDM2 oncogenic properties could come together with p53 reactivation should be seriously kept into account, unless specific strategies to limit these potential side effects are designed.

Last, but not least, the inborn leaning to develop multi-drug resistance brought about highly effective death-inducing approaches in most aggressive tumors, should be considered. Indeed, continuous exposure to Nutlin-3 promotes appearance of *de novo* p53 mutations associated with a multi-drug-resistant phenotype with an extremely high rate in several cancer cells, including neuroblastoma (Michaelis et al., 2011). Together with the notion that uncoupling MDM2–p53 interaction equally drives stabilization of wild type and mutant-p53 proteins, this may represent a very major concern to the straightforward application of MDM2-antagonists to cancer therapy, unless equally potent p53-independent approaches are developed and tested in combination therapy. An interesting opportunity may be offered by the high rate of replication stress of Myc-driven tumors (including MNA neuroblastoma) and by their consequent high sensitivity to inhibitors of ATR and CHK1 (Murga et al., 2011), two main signal transducers in the DNA replication stress response. Importantly, cell death induced by ATR or CHK1 inhibitors occurs independent of p53, suggesting that reactivation of p53 and inhibition of the replication stress response might trigger distinct cell death pathways in Myc-driven tumors. Based on this, it appears possible that the careful design of therapeutic strategies and administration schedules aimed at attacking cancer cells on distinct, biologically relevant pathways, such as DDR and ARF/MDM2/p53 circuitry, might be highly effective and at the same time able to circumvent development of biological resistances in order to significantly contribute to defeat MNA neuroblastoma tumors.

## Conflict of Interest Statement

The authors declare that the research was conducted in the absence of any commercial or financial relationships that could be construed as a potential conflict of interest.
